# Análisis de concordancia de tres pruebas para el diagnóstico de malaria en la población sintomática de los municipios endémicos de Colombia

**DOI:** 10.7705/biomedica.4893

**Published:** 2020-03-30

**Authors:** Liliana Jazmín Cortés, Ángela Patricia Guerra

**Affiliations:** 1 Grupo de Parasitología, Subdirección Laboratorio Nacional de Referencia, Dirección de Redes en Salud Pública, Instituto Nacional de Salud, Bogotá, D.C., Colombia Instituto Nacional de Salud BogotáD.C Colombia

**Keywords:** malaria/diagnóstico, microscopía, reacción en cadena de la polimerasa, Colombia, Malaria/diagnosis, microscopy, polymerase chain reaction, Colombia

## Abstract

**Introducción.:**

Dadas las dificultades del diagnóstico microscópico de la malaria o paludismo en las áreas rurales, las pruebas de diagnóstico rápido constituyen una buena alternativa, por lo que es importante conocer su desempeño.

**Objetivo.:**

Evaluar el desempeño de las pruebas de diagnóstico rápido utilizadas en cinco departamentos para al diagnóstico microscópico de la malaria usando la reacción en cadena de la polimerasa (PCR) como estándar de referencia.

**Materiales y métodos.:**

Se usaron la prueba de gota gruesa y las pruebas de diagnóstico rápido y, además, se impregnó papel de filtro con sangre para la prueba molecular (PCR), en individuos sintomáticos.

**Resultados.:**

Se incluyeron 314 muestras cuyo porcentaje de positividad para malaria fue de 49 % con la PCR, de 48 % con microscopía y de 46 % con las pruebas de diagnóstico rápido; la parasitemia fluctuó entre 180 y 23.800 parásitos/pl de sangre.

La concordancia de los resultados de los puestos de microscopía comparados con la PCR (Laboratorio Nacional de Referencia) fueron los siguientes: coeficiente kappa de Cohen de 0,975 (IC_95_% 0,950-0,999), sensibilidad de 97 % (IC_95_% 95-100) y especificidad de 100 % (IC_95_% 100-100), e índice kappa de especie de 0,958 (IC_95_% 0,912-1,00).

La concordancia de los resultados de la prueba de diagnóstico rápido Pf/Pv en los puestos de microscopía y los de la PCR (Laboratorio Nacional de Referencia), fue la siguiente: coeficiente kappa de 0,878 (IC_95_% 0,784-0,973), sensibilidad de 94 % (IC_95_% 87-100), especificidad de 95 % (IC_95_% 90-100), e índice kappa de especie de 1,0 (IC_95_% 1,00-1,00).

La concordancia entre la prueba de diagnóstico rápido Pf/Pan y la PCR fue la siguiente: coeficiente kappa de Cohen de 0,920 (IC_95_% 0,865-0,974), sensibilidad de 94 % (IC_95_% 90-98), especificidad de 99 % (IC_95_% 95-100), e índice kappa de especie de 0,750 (IC_95_% 0,637-0,863).

**Conclusión.:**

Los resultados de este estudio respaldan el uso de las pruebas de diagnóstico rápido en Colombia, aunque se requiere un mejor entrenamiento del personal para diferenciar eficientemente las especies de *Plasmodium.*

## Abstract (en)

**Introduction:**

Taking into account the difficulty of performing malaria microscopic diagnosis in rural areas, rapid diagnostic tests (RDT) are a good alternative, but it is important to verify their diagnostic performance.

**Objective:**

To evaluate the diagnostic performance of the RDTs used in five Colombian departments by comparing them with the microscopic diagnosis and using PCR as the reference standard.

**Materials and methods:**

Thick blood film and RDTs were used to diagnose symptomatic individuals; additionally, the filter paper was impregnated with blood for the molecular test.

**Results:**

We included 314 samples whose percentage of positivity for malaria was 49% by PCR, 48% by microscopy and 46% by RDT; parasitemia ranged between 180 and 23,800 p/µl of blood.

The concordance of the results from the microscopy units and those of the PCR (National Laboratory of Reference) was as follows: Cohen's kappa coefficient, 0.975 (95% CI: 0.9500.999); sensitivity, 97% (95% CI 95-100); specificity 100% (95% CI: 100-100), and kappa index of species, 0.958 (IC95%: 0.912-1.00).

The concordance between the Pf/Pv RDT (at the microscopy units) and the PCR (National Laboratory of Reference) was as follows: kappa coefficient, 0.878 (95% CI: 0.784-0.973); sensitivity, 94% (95% CI: 87-100); specificity, 95% (95% CI: 90-100), and kappa index of species, 1.0 (95% CI: 1.00-1.00).

The concordance between the Pf/Pan RDT versus PCR was: Cohen's kappa coefficient, 0.920 (95 % CI: 0.865- 0.974); sensitivity, 94% (95% CI: 90-98); specificity, 99% (95% CI 95-100), and kappa index of species, 0.750 (IC95% 0,637-0,863).

**Conclusion:**

The results of this study support the use of RDTs in Colombia; however, more training of the personnel is required to accurately differentiate *Plasmodium* species.

En Colombia, la malaria continúa siendo un grave problema de salud pública. En el 2016, fue el segundo país con mayor aumento en el número de casos de malaria en las Américas (27.942 casos más que en el 2015), principalmente debidos a la infección por *Plasmodium falciparum* (1).

Entre las posibles causas de este incremento, se encuentra el fenómeno de El Niño-Oscilación Sur y la colindancia geográfica entre Colombia y Venezuela, situación que actualmente ha favorecido el desplazamiento transfronterizo de personas infectadas desde territorio venezolano ^1^.

En el 2017, se presentó una disminución del 35 % en la notificación de los casos de malaria con respecto al 2016, y fueron los departamentos de Chocó, Nariño, Antioquia, Córdoba, Guainía, Amazonas, Cauca y Vichada los que aportaron el mayor número de casos ^2^. En ese año, se registraron 55.117 casos, de los cuales 54.102 fueron de malaria no complicada y 1.015 de malaria complicada, además, hubo 19 muertes ^2^.

Frente a este panorama de transmisión de la enfermedad, es necesario contar con un diagnóstico oportuno y de calidad que permita una atención adecuada de los pacientes y la administración oportuna y eficaz del tratamiento.

Los dos métodos usados rutinariamente para el diagnóstico de la malaria son la microscopía de luz y las pruebas de inmunocromatografía o pruebas de diagnóstico rápido ^3^. Actualmente, la microscopia constituye el método estándar de referencia para el diagnóstico de rutina de la malaria; el umbral de detección de la gota gruesa es de 10 a 30 parásitos/microlitro (p/µl) de sangre, lo que equivale aproximadamente a 0,001 % de glóbulos rojos infectados ^4^. Otros autores han registrado una sensibilidad del 98,36 % en un estudio en el que se comparó la sensibilidad de la gota gruesa con la del extendido y con un método de detección de antígeno ^5^. Sin embargo, en condiciones de campo, un umbral de 50 a 100 p/µl de sangre es más realista ^6^^,^^7^.

La gota gruesa se considera como la prueba de referencia, ya que permite determinar la densidad parasitaria, diferenciar especies y estadios del parásito, hacer seguimiento a la respuesta al tratamiento, detectar gametocitos, diagnosticar otros parásitos de la sangre y controlar la calidad de la microscopía. Por otro lado, las pruebas de diagnóstico rápido permiten detectar antígenos específicos del parásito con una sensibilidad de 95 % a partir de ~100 p/µl de sangre ^4^ y constituyen una herramienta complementaria en aquellas áreas en donde no es posible acceder al diagnóstico microscópico o en situaciones de brote.

Las pruebas más utilizadas se basan en la detección de la proteína II rica en histidina (HRP-2), con la cual solo se identifica *P. falciparum,* y en la detección de la deshidrogenasa de lactato parasitaria (pLDH) o de la p-aldolasa, isoenzimas producidas por el parásito durante el ciclo eritrocítico, con las cuales se identifican todas las especies ^8^. Una de las principales desventajas de dichas pruebas son los resultados falsos positivos debido a la persistencia del antígeno HRP-2 en la sangre durante varios días después de la eliminación de la infección por acción del tratamiento antimalárico ^9^^,^^10^ y, los falsos negativos, debido a la deleción del gen *HRP2,* como se ha visto en aislamientos de varias partes del mundo ^11^.

El propósito de este estudio fue evaluar en campo el desempeño diagnóstico de las pruebas rápidas de diagnóstico suministradas por el Ministerio de Salud durante el 2016 y el 2017 a las secretarías departamentales de salud y compararlo con el diagnóstico microscópico mediante la reacción en cadena de la polimerasa (PCR) anidada como estándar de referencia.

## Materiales y métodos

### Población de estudio

La población de estudio estuvo constituida por individuos de ambos sexos y diferentes edades residentes en cinco departamentos endémicos para malaria, que acudieron al servicio de salud con síntomas como fiebre, escalofríos, malestar general y cefalea, entre otros, en busca de un diagnóstico.

Para el cálculo del tamaño de la muestra, se consideró la prevalencia de casos por municipio con una confianza del 95 %, con lo que la muestra se estableció en 398 pacientes de los cinco departamentos. El muestreo fue por conveniencia hasta completar el número calculado.

A cada individuo se le hicieron la prueba de la gota gruesa y la de diagnóstico rápido, y se tomó una muestra de sangre en papel de filtro. La lectura de la gota gruesa y la prueba de diagnóstico rápido la hizo en el sitio de diagnóstico, un microscopista con experiencia en ambas pruebas por su trabajo de atención rutinaria de pacientes. A estos se les administró el tratamiento correspondiente y sus casos se notificaron al Sivigila. Posteriormente, las láminas con las gotas gruesas, los dispositivos de las pruebas rápidas de diagnóstico y las tarjetas de papel de filtro de cada paciente, fueron remitidos al Grupo de Parasitología del Laboratorio Nacional de Referencia del Instituto Nacional de Salud, donde se hicieron las pruebas moleculares y se verificó la lectura de las gotas gruesas y de las pruebas rápidas.

La fase de recolección de muestras se limitó al tiempo que tomó alcanzar el tamaño de muestra proyectado para cada municipio, entre 2 y 4 meses. Los criterios tenidos en cuenta para la recolección del material y su correspondiente envío y control de calidad en el Laboratorio Nacional de Referencia, fueron: a) el envío del 50 % de las muestras de pacientes positivos y el 50 % de los pacientes negativos, y b) inclusión en las muestras positivas de los casos diagnosticados como malaria por *P. vivax* o por *P. falciparum, así* como las infecciones mixtas.

### Diagnóstico microscópico

El diagnóstico microscópico se hizo en los puestos de microscopía de los cinco departamentos mediante la técnica de la gota gruesa y la coloración con tinción modificada de Romanowsky; se reportó la positividad, la especie y el recuento parasitario, siguiendo los lineamientos nacionales ^12^.

### Diagnóstico mediante las pruebas de diagnóstico rápido

En los departamentos de Chocó, Guainía y Cauca, se utilizaron las pruebas Ag Pf/Pan y, en Nariño y el distrito de Buenaventura, las pruebas Ag Pf/Pv, distribuidas por el Ministerio de Salud y Protección Social.

Las primeras (Pf/Pan) contienen una tira de membrana previamente recubierta con anticuerpos monoclonales específicos contra la HRP-2 de *P. falciparum* en la región Pf de la línea de prueba, y con anticuerpos monoclonales específicos contra la deshidrogenasa de lactato de *P. falciparum, P. vivax, P. malariae* y *P. ovale* en la región Pan de la línea de prueba. Las segundas (Pf/Pv) contienen una tira de membrana previamente recubierta con anticuerpos monoclonales específicos contra la HRP-2 de *P. falciparum* en la región Pf de la línea de prueba, y con anticuerpos monoclonales específicos contra la deshidrogenasa de lactato de *P. vivax* en la región Pv de la línea de prueba. Las pruebas se realizaron y se interpretaron siguiendo las instrucciones del fabricante.

Los dos tipos de pruebas rápidas empleadas son las recomendadas por la Organización Mundial de la Salud (OMS) a partir de la evaluación anual que realiza la *Foundation for Innovative New Diagnostics* (FIND) (ronda 2015-2016). No se hizo una evaluación previa de la sensibilidad ni de la especificidad de estas pruebas.

### Control de calidad de las pruebas de gota gruesa

El Laboratorio Nacional de Referencia hizo el control de calidad de la lectura de las gotas gruesas reportadas por los puestos de microscopía según los lineamientos nacionales ^12^. Una vez finalizada la lectura de las láminas, se compararon los resultados obtenidos en los puestos de microscopía con los del Instituto Nacional de Salud, se seleccionaron las láminas discordantes y, con el fin de evitar sesgos, se ocultó su identificación para que el mismo lector las revisara nuevamente en ciego.

### Control de calidad de la interpretación de las pruebas rápidas de diagnóstico por parte del Laboratorio Nacional de Referencia

El Laboratorio Nacional de Referencia hizo el control de calidad de la interpretación de las pruebas rápidas reportadas por los puestos de microscopía siguiendo las instrucciones del fabricante y en ciego frente a los resultados de la microscopía y la PCR. Una vez finalizada la lectura de los casetes, se compararon los resultados obtenidos en los puestos de microscopía con los del Instituto Nacional de Salud.

### Extracción de ADN del parásito y amplificación por PCR

El ADN del parásito se extrajo a partir de las muestras de sangre impregnadas en papel de filtro, usando el estuche de purificación de ADN genómico QiaAmp DNA Micro™ (Qiagen) y siguiendo las recomendaciones del fabricante.

Para la detección e identificación de *P. vivax* y *P. falciparum,* se usó una PCR anidada para amplificar regiones muy conservadas del gen que codifica para la subunidad pequeña del ARN ribosómico. En la primera reacción de PCR se utilizaron cebadores específicos de género (rPLU6 y rPLU5), según las condiciones descritas por Snounou, *et al.*^13^. Para la segunda PCR anidada, se utilizaron las parejas de cebadores rFAL1 y rFAL2 y rVIV1 y rVIV2, específicos de especie, para amplificar *P. falciparum* y *P. vivax,* respectivamente, siguiendo el protocolo descrito por Singh, *et al.*^14^.

En la primera PCR, la mezcla de reacción incluyó 1 U *deTaq* ADN polimerasa (Promega), 2 mM de MgCl_2_, 125 pM de trifosfato de desoxiadenosina (dNTP) (Invitrogen™) y 0,25 pM de cebadores (IDT™); como plantilla se usaron 5 pl de ADN. Las condiciones de la PCR fueron: desnaturalización inicial a 95 °C durante cinco minutos, anillamiento a 58 °C durante dos minutos, extensión a 72 °C durante dos minutos, desnaturalización a 94 °C durante un minuto con 25 ciclos de repetición entre los pasos 2 y 4, seguida de anillamiento a 58 °C durante dos minutos y una extensión final a 72 °C durante cinco minutos.

Para la PCR anidada, la mezcla de reacción consistió en 0,5 U de *Taq* ADN polimerasa (Promega), 2 mM de MgCl_2_, 200 pM de dNTP, 0,25 pM de cebadores y 1 pl del amplificado anterior como plantilla. Las condiciones del termociclador fueron: desnaturalización inicial a 94 °C durante cuatro minutos, desnaturalización a 94 °C durante 30 segundos, anillamiento a 58 °C durante un minuto y extensión a 72 °C durante un minuto, con 30 ciclos de repetición entre los pasos 2 y 4 seguidos de una extensión final a 72 °C durante cuatro minutos.

Los productos obtenidos en la PCR anidada se sometieron a electroforesis en gel de agarosa al 2 % con Gel Red™. Las bandas se visualizaron bajo luz ultravioleta y se analizaron usando el programa ImageLab™. El tamaño de los fragmentos esperados fue de 205 pb para *P. falciparum* y de 120 pb para *P. vivax.*

### Análisis estadísticos

Se creó una base de datos en Excel con los resultados de las tres pruebas, y se calcularon el coeficiente kappa de Cohen, el índice kappa de especie, la sensibilidad y la especificidad.

### Consideraciones éticas

Este estudio se enmarca en la vigilancia por laboratorio en salud pública, por lo que se tomaron las muestras en papel de filtro, con el fin de comparar las técnicas de diagnóstico, sin que ello alterara el diagnóstico o el tratamiento oportuno de los pacientes en el lugar de la toma de la muestra. Los individuos fueron atendidos en el puesto de salud en la medida en que solicitaron el diagnóstico y recibieron el respectivo tratamiento según la guía de atención de la malaria vigente a nivel nacional. Los pacientes fueron invitados a participar en el estudio y se incluyeron los que aceptaron

## Resultados

En cuatro de los 25 municipios seleccionados no se tomaron muestras y en otros cuatro se tomaron menos de las solicitadas. Los municipios que no enviaron muestras (37 de las muestras programadas) fueron: Cantón de San Pablo, Istmina y Nóvita, en Chocó, y Pizarro, en Nariño. Otros municipios como Bagadó, Tadó, Quibdó y Barbacoas recolectaron 26 muestras menos de las programadas.

El número total de muestras recibidas en el laboratorio fue de 335 (cuadro 1). Sin embargo, de estas, 21 llegaron incompletas, es decir, no llegó la lámina, el papel de filtro o el casete de la prueba de diagnóstico rápido. Los análisis se hicieron únicamente con aquellas muestras que contaban con los tres resultados, es decir, 314 muestras.

**Cuadro 1:** Tamaño de muestra por departamento

Departamento	Municipio	Tamaño estimado de muestra	Prueba de diagnóstico rápido utilizada	Muestras recibidas en el laboratorio
Chocó	Atrato	8	Pf/Pan	196
(n=254)	Bagadó	26		
	Cantón de San Pablo	10		
	Condoto	5		
	Istmina	12		
	Lloró	26		
	Medio Atrato	9		
	Medio Baudó (Boca de Pepe)	11		
	Nóvita	10		
	Quibdó	99		
	Rioquito	13		
	Tadó	25		
Nariño	Barbacoas	13	Pf/Pv	89
(n=94)	El Charco	12		
	Maguí	5		
	Mosquera	5		
	Olaya Herrera	8		
	Pizarro	5		
	Roberto Payán	15		
	Santa Bárbara	5		
	Tumaco	26		
Guainía	Inírida	16	Pf/Pan	16
(n=16)				
Cauca	Guapi	5	Pf/Pan	10
(n=10)	Timbiquí	5		
Valle	Buenaventura	24	Pf/Pv	24
(n=24)				
Total		398		335

De los 314 individuos que aceptaron ser incluidos en el estudio, el 58,3 % correspondía a hombres y el 41,7 % a mujeres. El porcentaje de muestras positivas para malaria fue de 49 % por PCR, de 48 % por microscopía y de 46 % por la prueba rápida (cuadro 2).

**Cuadro 2:** Resultados según prueba diagnóstica

Resultado de las muestras	PCR (n)	Gota gruesa (n)	Prueba de diagnóstico rápido Pf/Pv (n)	Prueba de diagnóstico rápido Pf/Pan (n)
Infección por *Plasmodium falciparum*	97	94	32	45
Infección por *Plasmodium vivax*	56	53	2	50
Infección mixta	1	3	16	0
Positivas	154 (49 %)	150 (48 %)	145 (46 %)	
Negativas	160	164	169	
Analizadas	314	314	314	

Según los resultados de microscopía, la parasitemia estuvo entre 180 y 999 p/µl en 23,8 % de los pacientes, entre 1.000 y 1.999 p/µl en el 10,5 %, entre 2.000 y 4.999 p/µl en el 30,5 %, entre 5.000 y 9.999 p/µl en el 20 % y entre 10.000 y 14.999 p/µl en el 11,4 %. Solo el 3,8 % de los pacientes tuvo una parasitemia de menos de 15.000 p/µl, en tanto que la más alta encontrada fue de 23.800 p/µl. Del grupo con la parasitemia más baja, el 12,4 % de los casos estuvo por debajo de 500 p/µl, y la más baja fue de 180 p/µl. La mediana de la parasitemia fue de 3.200 p/µl.

### Concordancia de los resultados de las pruebas de gota gruesa realizadas en los puestos de microscopía y la PCR

Se obtuvo un coeficiente kappa de Cohen de 0,975 (IC_95_% 0,950-0,999). Contrastada con la prueba de referencia (PCR), la gota gruesa tuvo una sensibilidad de 97 % (IC_95_% 95-100) y una especificidad del 100 % (IC_95_% 100-100) (cuadro 3). Se calculó el índice kappa de especie incluyendo 150 láminas positivas y se obtuvo un valor de 0,958 (IC_95_% 0,912-1,00); además, la sensibilidad fue de 95 % (IC_95%_ 89-100) para *P. vivax,* de 97 % (IC_95%_ 93100) para *P. falciparum* y de 100 % (IC_95%_ 100-100) para la infección mixta.

**Cuadro 3:** Comparación de los resultados de la PCR y la gota gruesa

Prueba	PCR			
	Resultado	Positiva	Negativa	Total
Gota gruesa	Positivo	150	0	154
	Negativo	4	160	164
Total		154	160	314

Índice kappa=0,975 (IC_95_% 0,950-0,999)

Sensibilidad=97 % (IC_95_% 95-100)

Especificidad=100 % (IC_95_% 100-100)

### Concordancia de la prueba rápida Pf/Pv realizada en los puestos de microscopía y la PCR

Se obtuvo un coeficiente kappa de Cohen de 0,878 (IC_95_% 0,784-0,973). Contrastada con la prueba de referencia (PCR), esta prueba rápida tuvo una sensibilidad de 94 % (IC_95%_ 87-100) y una especificidad del 95 % (IC_95%_ 90-100) (cuadro 4). Se calculó el índice kappa de especie incluyendo 34 pruebas rápidas positivas y se obtuvo un valor de kappa de 1,0 (IC_95_% 1,001,00); además, una sensibilidad de 100 % (IC_95_% 100-100) para *P. vivax* y una de 100 % (IC_95%_ 100-100) para *P. falciparum,* en tanto que no hubo muestras con diagnóstico de infección mixta.

**Cuadro 4:** Comparación de los resultados de la PCR y la prueba rápida Pf/Pv

Prueba	PCR			
	Resultado	Positiva	Negativa	Total
Gota gruesa	Positivo	34	4	38
	Negativo	2	71	73
Total		36	75	111

Índice kappa=0,878 (IC_95_% 0,784-0,973)

Sensibilidad=94 % (IC_95_% 87-100)

Especificidad=95 % (IC_95%_ 90-100)

### Concordancia de la prueba rápida Pf/Pan realizada en los puestos de microscopía y la PCR

Se obtuvo un coeficiente kappa de Cohen de 0,920 (IC_95_% 0,865-,0974). Contrastada con la prueba de referencia (PCR), esta prueba rápida tuvo una sensibilidad de 94 % (IC_95_% 90-98) y una especificidad de 99 % (IC_95_% 97-100) (cuadro 5). Se calculó el índice kappa de especie incluyendo 111 pruebas positivas y se obtuvo un valor de 0,750 (IC_95_% 0,637-0,863); además, una sensibilidad de 98 % (IC_95%_ 94-100) para *P. vivax,* de 76 % (IC_95%_ 65-87) para *P. falciparum,* y de 6 % (IC_95%_ 6-18) para la infección mixta.

**Cuadro 5:** Comparación de los resultados de la PCR y de la prueba rápida Pf/Pan

Prueba	PCR			
	Resultado	Positiva	Negativa	Total
Gota gruesa	Positivo	111	1	112
	Negativo	7	84	91
Total		118	85	203

Índice kappa=0,920 (IC_95%_ 0,865-0,974)

Sensibilidad=94 % (IC_95%_ 90-98)

Especificidad=99 % (IC_95%_ 97-100)

### Concordancia de la prueba rápida Pf/Pv y la evaluación por microscopía realizada en los puestos de microscopía

Se obtuvo un coeficiente kappa de Cohen de 0,897 (IC_95_% 0,808-0,985). Contrastada con la prueba de referencia (gota gruesa), esta prueba rápida tuvo una sensibilidad de 100 % (IC_95%_100-100) y una especificidad de 94 % (IC_95%_ 88-99).

### Concordancia de la prueba rápida Pf/Pan y la evaluación por microscopía realizada en los puestos de microscopía

Se obtuvo un coeficiente kappa de Cohen de 0,930 (IC_95_% 0,879-0,981). Contrastada con la prueba de referencia (gota gruesa), esta prueba rápida tuvo una sensibilidad de 95 % (IC_95%_ 91-99) y una especificidad de 99 % (IC95% 97-100).

### Control de calidad realizado por el Laboratorio Nacional de Referencia

Se compararon los resultados de la microscopía del Laboratorio Nacional de Referencia con los obtenidos en el puesto de microscopía. La concordancia de los positivos fue de 99 % (IC_95%_ 97-100) y la de los negativos de 100 % (IC_95%_100-100); el coeficiente kappa de Cohen fue de 0,987 (IC_95%_ 0,970-1,00). Se calculó el índice kappa de especie incluyendo 150 láminas positivas y se obtuvo un valor de 0,945 (IC_95_% 0,891-0,998).

### Interpretación de las pruebas rápidas en el LNR Vs. las realizadas en los puestos de microscopía

Se compararon los resultados de la la prueba rápida Pf/Pv del Laboratorio Nacional de Referencia con los obtenidos en el puesto de microscopía. Se obtuvo un coeficiente kappa de Cohen de 0,959 (IC_95_% 0,904-1,015); el índice kappa de especie se calculó incluyendo 36 pruebas rápidas positivas y se obtuvo un valor de 1,0 (IC_95_% 1,00-1,00).

Se compararon los resultados de la la prueba rápida Pf/Pan del Laboratorio Nacional de Referencia con los obtenidos en el puesto de microscopía. Se obtuvo un coeficiente kappa de Cohen de 1,00 (IC_95_% 1,001,00); el índice kappa de especie se calculó incluyendo 112 pruebas positivas y se obtuvo un valor de 0,752 (IC_95_% 0,639-0,864).

### Sensibilidad de las pruebas rápidas según la parasitemia

Se obtuvo una sensibilidad de 84 % (IC_95%_ 68-100) con una parasitemia de menos de 999 p/µl, de 91 % (IC_95_% 81-100) con una entre 1.000 y 4.999 p/ pl, de 100 % (IC_95_% 100-100) con una entre 5.000 y 9.999 p/µl, de 89 % (IC_95_% 68-100) con una entre 10.000 y 14.999 p/µl, y de 100 % (IC_95_% 100-100) con ° una de más de 14.999 p/µl.

*Prueba rápida Pf/Pv.* La sensibilidad fue de 33 % (IC_95%_ 4-71) con una parasitemia de menos de 999 p/µl y alcanzó el 100 % (IC_95%_ 100-100) en los otros rangos (1.000 a 4.999, 5.000 a 9.999, 10.000 a 14.999 y más de 14.999 p/µl).

## Discusión

La Organización Panamericana de la Salud (OPS) planteó coordinar los esfuerzos para controlar y eliminar la malaria en la región de las Américas mediante el 'Plan de acción para la eliminación de la malaria, 2016-2020' ^15^, el cual se enfoca en las herramientas, actividades y estrategias necesarias para lograr la interrupción de la transmisión, las cuales deben ser adaptadas localmente a los planes estratégicos nacionales de eliminación ^16^.

Desde la perspectiva de disminuir el número de casos de malaria, es importante contar con un diagnóstico oportuno y adecuado, que actualmente se basa en la microscopía como técnica de referencia. Sin embargo, el uso de pruebas de diagnóstico rápido de la malaria se ha venido extendiendo a nivel nacional, por lo cual se han dado recomendaciones para utilizarlas en zonas geográficas en las que no se hace el diagnóstico microscópico o para los casos de brote de la enfermedad en que debe brindarse un diagnóstico oportuno a toda la población afectada mediante estrategias de búsqueda activa ^12^. Las pruebas rápidas se han convertido en un componente esencial de los esfuerzos para mejorar la precisión del diagnóstico en aquellas áreas donde una gran parte de la población está en riesgo de contraer la malaria y son cada vez más importantes en el manejo, el control y la eliminación de los casos ^17^.

En este contexto, se planteó evaluar las pruebas rápidas comparándolas con la prueba de referencia, es decir, la PCR, teniendo en cuenta las especificaciones técnicas según los antígenos parasitarios que detectan. El coeficiente kappa de Cohen de las pruebas Pf/Pan fue de 0,920 (IC_95_% 0,8650,0974); debe tenerse en cuenta que se registraron un falso positivo y siete falsos negativos en esta prueba pues, de las 203 muestras analizadas, siete fueron positivas en la PCR y negativas en la prueba rápida, y una muestra fue negativa en la PCR y positiva en la prueba rápida. Estos resultados estarían relacionados con la especie y la densidad parasitaria, ya que la sensibilidad fue de 84 % (IC_95_% 68-100) en las muestras con una parasitemia de menos de 999 p/µl (cuadro 6).

**Cuadro 6:** Sensibilidad de las pruebas de diagnóstico rápido por rangos de parasitemia

Parasitemia (rangos)	Sensibilidad de la prueba (IC_95%_)	
	Pf/Pv	Pf/pan
<999	33 % (IC_95%_ 40-71)	84 % (IC_95%_ 60-95)
1.000-4.999	100 % (IC_95%_ 100-100)	91 % (IC_95%_ 76-99)
5.000-9.999	100 % (IC_95%_ 100-100)	100 % (IC_95%_ 100-100)
10.000-14.999	100 % (IC_95%_ 100-100)	89 % (IC_95%_ 68-100)
>15.000	100 % (IC_95%_ 100-100)	100 % (IC_95%_ 100-100)

En cuanto a la especie parasitaria, el índice kappa de especie fue de 0,750 (IC_95_% 0,637-0,863), lo que refleja el inadecuado diagnóstico como infección mixta en una muestra diagnosticada como *P. vivax* en la PCR, y en otras 14 que se diagnosticaron como *P. falciparum* con la PCR. Este resultado estaría relacionado con la recomendación de lectura del fabricante, según la cual la presencia de tres bandas de color puede interpretarse como infección por *P. falciparum* o como infección mixta, lo que no ocurre en las pruebas Ag Pf/Pv, que sí permiten diferenciar la infección mixta de las muestras positivas para *P. falciparum.* Por ello, en las recomendaciones técnicas del Laboratorio Nacional de Referencia para la compra de estas pruebas diagnósticas, se sugiere escoger una que incluya un anticuerpo monoclonal específico para *P. vivax* en vez del antígeno panmalárico que puede afectar la sensibilidad del diagnóstico en campo.

Hubo nueve muestras con resultado falso negativo en las pruebas rápidas. En estos casos, para comprobar una posible deleción del gen *hrp2,* este se amplificó mediante PCR, obteniéndose una banda a la altura esperada en cada muestra. La ausencia de señal en la prueba rápida de muestras con el gen *hrp2* estaría relacionada con diversos factores que influyen en su desempeño en campo, por ejemplo, su calidad, las condiciones de almacenamiento, la capacidad del usuario para interpretar correctamente el resultado y la densidad de parásitos, entre otros ^18^^-^^21^.

El índice kappa de la prueba de diagnóstico rápido Pf/Pv fue de 0,878 (IC_95_% 0,784-0,973), resultado que se explica por el diagnóstico de dos falsos negativos y cuatro falsos positivos, ya que de las 111 muestras analizadas, dos fueron positivas en la PCR y negativas en la prueba rápida, en tanto que cuatro fueron negativas en la PCR y positivas en la prueba rápida. Estos resultados estarían relacionados con la especie y la densidad parasitaria, ya que se obtuvo una sensibilidad del 33 % (IC_95%_ 30-83) en las muestras con una parasitemia de menos de 999 p/µl (cuadro 6).

En cuanto a la especie parasitaria, se calculó el índice kappa de especie obteniéndose un valor de 1,0 (IC_95_% 1,00-1,00). Sin embargo, debe tenerse en cuenta que en los sitios donde se emplearon las pruebas rápidas Pf/Pv no se diagnosticaron infecciones mixtas en las muestras seleccionadas.

Por otra parte, se evaluó la concordancia de la gota gruesa estudiada en los puestos de microscopía y la PCR, obteniéndose un coeficiente kappa de Cohen de 0,975, concordancias de positividad y negatividad de 97 y 100 %, respectivamente, y un índice kappa de especie de 0,958, lo que concuerda con los resultados obtenidos en el control de calidad de la lectura de las gotas gruesas de los puestos de microscopía por parte del Laboratorio Nacional de Referencia. Esto significa que la calidad del diagnóstico microscópico es buena.

En cuanto a los rangos de parasitemia, los valores de sensibilidad y especificidad de las pruebas rápidas se vieron influenciados por las condiciones de baja densidad parasitaria, con una sensibilidad del 33 % (IC_95_% 30-83) de la prueba rápida Pf/Pv en las muestras con una parasitemia de menos de 999 p/µl. Los demás resultados obtenidos fueron buenos a la luz de los criterios vigentes de evaluación de la OPS: sensibilidad de las pruebas de *P. falciparum* de ≥75 % con 200 p/µl y de las pruebas de *P. vivax* de ≥75 % con 200 p/µl; tasa de falsos positivos menor del 10 % con muestras negativas limpias y tasa de pruebas no válidas menor de 5 % ^22^.

Al comparar los resultados obtenidos con los de otros estudios, la sensibilidad de las pruebas rápidas comparada con la de la gota gruesa en los puestos de microscopía en el diagnóstico de ambas especies de *Plasmodium,* fue de 94 % y la especificidad osciló entre 95 (Pf/Pv) y 99 % (Pf/Pan). Estos resultados son muy similares a los de Montoya, *et al.,* quienes obtuvieron una sensibilidad de 93,85 % (IC_95_% 87,23-100) y una especificidad de 94,29 % (IC_95_% 85,17-100) ^23^.

En cuanto a la PCR comparada con la gota gruesa, Montoya, *et al.,* refieren una sensibilidad de 100 % (IC_95_% 99,23-100) y una especificidad de 97,14 % (IC_95_% 90,19-100). En el presente estudio, la PCR se usó como prueba de referencia y sus resultados reflejaron una sensibilidad casi idéntica (97 %) comparados con los de la gota gruesa.

Vale la pena destacar que, aunque los métodos moleculares como la PCR anidada ofrecen gran sensibilidad y especificidad y se consideran el estándar de referencia para el diagnóstico de malaria, en este estudio se observó que la sensibilidad de la microscopía (gota gruesa), prueba de diagnóstico de rutina en nuestro país, fue bastante alta en comparación con la PCR, aunque se presentaron cuatro falsos negativos por microscopía, lo que se explicaría por la presencia de una parasitemia por debajo del límite de detección de la prueba. Teniendo en cuenta que la parasitemia usual en los pacientes colombianos es baja, es importante destacar la excelente labor y capacidad técnica de los microscopistas, ya que lograron detectar densidades parasitarias cercanas al límite de detección (desde180 p/µl de sangre).

Entre las limitaciones del estudio debe considerase que, aunque el tamaño de muestra se calculó a partir de la prevalencia de casos por municipio, con una confianza del 95 % (398 pacientes entre los cinco departamentos), debido a situaciones particulares de los sitios de diagnóstico, en algunos casos se tomaba la gota gruesa pero no la muestra para el papel de filtro o la prueba rápida, por lo que solo se analizaron las 314 muestras que contaban con resultados para las tres pruebas diagnósticas.

## References

[1] Organización Panamericana de la Salud-Organización Mundial de la Salud Situación de la Malaria en la Región de las Américas, 2000-2016.

[2] Salas D (2017). Informe de evento malaria, Colombia.

[3] World Health Organization (2014). Policy brief on malaria diagnostics in low-transmission settings.

[4] Perandin F, Manca G, Piccolo A, Calderaro A, Galati L, Ricci L (2003). Identification of Plasmodium falciparum, P. vivax, P. ovale and P. malariae and detection of mixed infection in patients with imported malaria in Italy. New Microbiol.

[5] Dharaiya CM, Faldu BR, Patel HL (2017). Comparative evaluation of thin smear, thick smear and antigen detection test in the diagnosis of malaria. Indian J Pathol Oncol.

[6] World Health Organization (1988). Malaria diagnosis: Memorandum from a WHO meeting. Bull World Health Organ.

[7] Milne LM, Kyi MS, Chiodini PL, Warhurst DC (1994). Accuracy of routine laboratory diagnosis of malaria in the United Kingdom. J Clin Pathol.

[8] Moody A (2002). Rapid diagnostic test for malaria parasites.. Clin Microbiol.

[9] Ugah UI, Alo MN, Owolabi JO, Okata-Nwali OD, Ekejindu IM, Ibeth N (2017). Evaluation of the utility value of three diagnostic methods in the detection of malaria parasites in endemic area. Malar J.

[10] Berzosa P, de Lucio A, Romay-Barja M, Herrador Z, González V, García L (2018). Comparison of three diagnostic methods (microscopy, RDT, and PCR) for the detection of malaria parasites in representative samples from Equatorial Guinea. Malar J.

[11] Berhane A, Russom M, Bahta I, Hagos F, Ghirmai M, Uqubay S (2017). Rapid diagnostic tests to detect Plasmodium falciparum infections in Eritrea: An investigation of reported false negative RDT results. Malar J.

[12] Cortés LJ, Guerra AP (2017). Guía para la vigilancia por laboratorio de parásitos del género *Plasmodium* spp.

[13] Snounou G, Viriyakosol S, Zhu X, Jarra W, Pinheiro I, Rosario V (1993). High sensivity of detection of human malaria parasites by the use of nested polymerase chain reaction. Mol Biochem Parasitol.

[14] Singh B, Bobogare A, Cox-Singh J, Snounou G, Abdullah MS, Rahman HA (1999). A genus- and species-specific nested polymerase chain reaction malaria detection assay for epidemiologic studies. Am J Trop Med Hyg.

[15] Organización Panamericana de la Salud Plan de acción para la eliminación de la malaria 2016-2020.

[16] Organización Mundial de la Salud Estrategia Técnica Mundial contra la Malaria 2016-2030.

[17] Gamboa D, Ho MF, Bendezu J, Torres K, Chiodini PL, Barnwell JW (2010). A large proportion of P. falciparum isolates in the Amazon region of Perú lack pfhrp2 and pfhrp3: Implications for malaria rapid diagnostic tests. PLoS ONE.

[18] Chiodini PL, Bowers K, Jorgensen P, Barnwell JW, Grady KK, Luchavez J (2007). The heat stability of Plasmodium lactate dehydrogenase-based and histidine-rich protein 2-based malaria rapid diagnostic tests. Trans R Soc Trop Med Hyg.

[19] Harvey SA, Jennings L, Chinyama M, Masaninga F, Mulholland K, Bell DR (2008). Improving community health worker use of malaria rapid diagnostic tests in Zambia: Package instructions, job aid and job aid-plus-training. Malar J.

[20] Rennie W, Phetsouvanh R, Lupisan S, Vanisaveth V, Hongvanthong B, Phompida S (2007). Minimizing human error in malaria rapid diagnosis: Clarity of written instructions and health worker performance. Trans R Soc Trop Med Hyg.

[21] Bell DR, Wilson DW, Martin LB (2005). False-positive results of a Plasmodium falciparum histidine-rich protein 2-detecting malaria rapid diagnostic test due to high sensitivity in a community with fluctuating low parasite density. Am J Trop Med Hyg.

[22] World Health Organization Anuncio público dirigido a los fabricantes de pruebas de diagnóstico rápido, organismos de adquisición y programas nacionales de control del paludismo.

[23] Montoya AE, Menco J, Osorio N, Zuluaga M, Duque J, Torres G (2008). Concordancia entre gota gruesa, inmunocromatografía y reacción en cadena de la polimerasa para el diagnóstico de malaria. Biomédica.

